# Supplementary material for: CROS or hearing aid? Selecting the ideal solution for unilateral CI patients with limited aidable hearing in the contralateral ear
Source: PLoS One. 2024 Feb 23;19(2):e0293811. doi: 10.1371/journal.pone.0293811 (PMC10890777; doi:10.1371/journal.pone.0293811)
Supplement: S1 File — (ZIP) [file pone.0293811.s002.zip › Baseline Needs Assessment.pdf]

Recipients with limited bimodal benefit: Hearing Aid or CROS
Baseline Hearing Needs Assessment

Subject-ID: ☐☐

Date: ☐☐ . ☐☐ . ☐☐☐☐
Month Day Year

The following questions are regarding your listening needs with your current hearing technology (i.e. your cochlear implant, with or without a hearing aid on the opposite ear).

Please rate your agreement with the following statements on a scale of 1 to 7, where 1 is extremely disagree and 7 is extremely agree:

1. I need to hear sounds from my non-CI side.

1	2	3	4	5	6	7
Extremely Disagree			Neutral			Extremely Agree

2. If a speaker is to the side, I prefer to place them in my CI side.

1	2	3	4	5	6	7
Extremely Disagree			Neutral			Extremely Agree

3. I think about where to sit during work or personal encounters based on my hearing needs.

1	2	3	4	5	6	7
Extremely Disagree			Neutral			Extremely Agree

4. I need to hear family/friends around the dinner table.

1	2	3	4	5	6	7
Extremely Disagree			Neutral			Extremely Agree

5. I need to hear other passengers in the car or other vehicles.

1	2	3	4	5	6	7
Extremely Disagree			Neutral			Extremely Agree

6. I need to hear other people in a large group.

1	2	3	4	5	6	7
Extremely Disagree			Neutral			Extremely Agree

7. I need to hear quiet voices better in quiet situations.

1	2	3	4	5	6	7
Extremely Disagree			Neutral			Extremely Agree

8. Please rank order the following listening situations based on how important they are to you. If not applicable, please say "N/A."

_____ Hearing family friends around the dinner table.

_____ Hearing other passengers in the car or other vehicles.

_____ Hearing other people in a large group.

_____ Hearing quiet voices better in quiet situations.

_____ Other, please specify: ____

_____ Other, please specify: ____
