# Supplementary material for: CROS or hearing aid? Selecting the ideal solution for unilateral CI patients with limited aidable hearing in the contralateral ear
Source: PLoS One. 2024 Feb 23;19(2):e0293811. doi: 10.1371/journal.pone.0293811 (PMC10890777; doi:10.1371/journal.pone.0293811)
Supplement: S1 File — (ZIP) [file pone.0293811.s002.zip › Chronic use_HA.pdf]

Recipients with limited bimodal benefit: Hearing Aid or CROS
Chronic Use of Study Hearing Aid

Subject-ID: ☐☐

Date: ☐☐ . ☐☐ . ☐☐☐☐
Month Day Year

In the following questions, we would like to understand how the study hearing aid contributes to your overall hearing experience with your cochlear implant.

Please rate your agreement with the following statements on a scale of 1 to 7, where 1 is extremely disagree and 7 is extremely agree:

1. The hearing aid helped me hear sounds from my non-CI side.

1	2	3	4	5	6	7
Extremely Disagree			Neutral			Extremely Agree

2. The hearing aid helped me think less about where to sit during work or personal encounters.

1	2	3	4	5	6	7
Extremely Disagree			Neutral			Extremely Agree

3. The hearing aid helped me hear family/friends around the dinner table.

1	2	3	4	5	6	7
Extremely Disagree			Neutral			Extremely Agree

4. The hearing aid helped me hear other passengers in the car or other vehicles.

1	2	3	4	5	6	7
Extremely Disagree			Neutral			Extremely Agree

5. The hearing aid helped me hear other people in a large group.

1	2	3	4	5	6	7
Extremely Disagree			Neutral			Extremely Agree

6. The hearing aid helped me hear quiet voices better in quiet situations.

1	2	3	4	5	6	7
Extremely Disagree			Neutral			Extremely Agree

7. The hearing aid helped me understand speech better.

1	2	3	4	5	6	7
Extremely Disagree			Neutral			Extremely Agree

8. The hearing aid improved the quality of sound.

1	2	3	4	5	6	7
Extremely Disagree			Neutral			Extremely Agree

9. The hearing aid improved the sound quality of music

1	2	3	4	5	6	7
Extremely Disagree			Neutral			Extremely Agree

10. I missed the hearing aid if I did not wear it for some reason.

1	2	3	4	5	6	7
Extremely Disagree			Neutral			Extremely Agree

11. The hearing aid provided me with a sense of balance.

1	2	3	4	5	6	7
Extremely Disagree			Neutral			Extremely Agree

12. The hearing aid helped me be more aware of sounds in my environment

1	2	3	4	5	6	7
Extremely Disagree			Neutral			Extremely Agree

13. The hearing aid made listening easier.

1	2	3	4	5	6	7
Extremely Disagree			Neutral			Extremely Agree

14. I was less tired at the end of day when listening with both- the hearing aid and cochlear implant, than with cochlear implant alone.

1	2	3	4	5	6	7
Extremely Disagree			Neutral			Extremely Agree

15. The hearing aid improved the quality of other people's voices.

1	2	3	4	5	6	7
Extremely Disagree			Neutral			Extremely Agree

16. The hearing aid helped me feel connected with the world.

1	2	3	4	5	6	7
Extremely Disagree			Neutral			Extremely Agree

17. The hearing aid enabled me to navigate my world more confidently.

1	2	3	4	5	6	7
Extremely Disagree			Neutral			Extremely Agree

18. The hearing aid made noisy situations more tolerable.

1	2	3	4	5	6	7
Extremely Disagree			Neutral			Extremely Agree

19. The hearing aid helped me turn around less to understand others during conversations.

1	2	3	4	5	6	7
Extremely Disagree			Neutral			Extremely Agree
