# Supplementary material for: CROS or hearing aid? Selecting the ideal solution for unilateral CI patients with limited aidable hearing in the contralateral ear
Source: PLoS One. 2024 Feb 23;19(2):e0293811. doi: 10.1371/journal.pone.0293811 (PMC10890777; doi:10.1371/journal.pone.0293811)
Supplement: S1 File — (ZIP) [file pone.0293811.s002.zip › Perceived Bimodal Benefit.pdf]

Recipients with limited bimodal benefit: Hearing Aid or CROS
Perceived Bimodal Benefit

Subject-ID: | | |

Date: | | | . | | | . | | | | |
Month Day Year

In the following questions, we would like to understand how **your** hearing aid contributes to your overall hearing experience with the cochlear implant.

Please rate your agreement with the following statements on a scale of 1 to 7, where 1 is extremely disagree and 7 is extremely agree:

1a. My hearing aid helps me understand speech better

1	2	3	4	5	6	7
Extremely Disagree			Neutral			Extremely Agree

1b. My hearing aid improves the quality of sound.

1	2	3	4	5	6	7
Extremely Disagree			Neutral			Extremely Agree

1c. My hearing aid improves the sound quality of music.

1	2	3	4	5	6	7
Extremely Disagree			Neutral			Extremely Agree

1d. I miss my hearing aid if I am not wearing it for some reason.

1	2	3	4	5	6	7
Extremely Disagree			Neutral			Extremely Agree

1e. My hearing aid provides me with a sense of balance.

1	2	3	4	5	6	7
Extremely Disagree			Neutral			Extremely Agree

1f. My hearing aid helps me be more aware of sounds in my environment.

1	2	3	4	5	6	7
Extremely Disagree			Neutral			Extremely Agree

1g. My hearing aid makes listening easier.

1	2	3	4	5	6	7
Extremely Disagree			Neutral			Extremely Agree

1h. I am less tired when listening with both- the hearing aid and cochlear implant, than with cochlear implant alone.

1	2	3	4	5	6	7
Extremely Disagree			Neutral			Extremely Agree
