# Supplementary material for: CROS or hearing aid? Selecting the ideal solution for unilateral CI patients with limited aidable hearing in the contralateral ear
Source: PLoS One. 2024 Feb 23;19(2):e0293811. doi: 10.1371/journal.pone.0293811 (PMC10890777; doi:10.1371/journal.pone.0293811)
Supplement: Attachment — Submitted filename: Response to Reviewers.docx [file pone.0293811.s004.docx]

September 27, 2023

Dear *PLOS ONE* Editorial Board,

We would like to express our sincere appreciation to the editor and the two anonymous reviewers for their invaluable time, expertise, and thoughtful insights in reviewing our manuscript titled *"CROS or Hearing Aid? Selecting the ideal solution for unilateral CI patients with limited aidable hearing in the contralateral ear."* The constructive feedback and meticulous attention to detail provided by the reviewers have played a pivotal role in refining the content and overall quality of our manuscript.

In the pages that follow, we address each comment from the editor and reviewers in an itemized fashion. Additionally, all suggestions have been implemented with changes tracked and can be found in the Word document titled “Revised Manuscript with Track Changes.”

Since the original submission, we have also addressed the following:

- Our study’s minimal underlying data set has been uploaded as a supporting information file (File: S1_Dataset).
- We included additional citations. One for the SSQ questionnaire, addressing an additional study limitation highlighted by one of the reviewers; and citations that reference the R packages that were required to carry out additional requested analyses.
- We have rectified a broken hyperlink in reference #27. All remaining citations are accurate and comprehensive.

In closing, we would like to confirm our adherence to all PLOS ONE policies on sharing data and materials by including the following updated Competing Interests section statement:

"I have read the journal's policy and the authors of this manuscript have the following competing interests: RD, CH, and SA are employees of Advanced Bionics. The remaining authors declare no conflicts of interest. This does not alter our adherence to PLOS ONE policies on sharing data and materials.”

If you have any additional questions for me, please feel free to connect with me at [sarah.lively@jefferson.edu](mailto:sarah.lively@jefferson.edu).

Sincerely,

Dr. Sarah Lively, Au.D.

Doctor of Audiology

[Sarah.Lively@Jefferson.Edu](mailto:Sarah.Lively@Jefferson.Edu)

**Editor comments to the author**

When submitting your revision, we need you to address these additional requirements.

1. Please ensure that your manuscript meets PLOS ONE's style requirements, including those for file naming. The PLOS ONE style templates can be found at

<https://journals.plos.org/plosone/s/file?id=wjVg/PLOSOne_formatting_sample_main_body.pdf>

and

<https://journals.plos.org/plosone/s/file?id=ba62/PLOSOne_formatting_sample_title_authors_affiliations.pdf>

**Author response**: Thank you for providing the links to PLOS ONE’s style requirements. We have reviewed them and applied the applicable edits with track changes.

2. We note that the grant information you provided in the ‘Funding Information’ and ‘Financial Disclosure’ sections do not match.

When you resubmit, please ensure that you provide the correct grant numbers for the awards you received for your study in the ‘Funding Information’ section.

**Author response**: We have updated the Funding Information and Financial Disclosures sections to match.

3. Thank you for stating the following in the Competing Interests section:

"I have read the journal's policy and the authors of this manuscript have the following competing interests: RD, CH, and SA are employees of Advanced Bionics. The remaining authors declare no conflicts of interest. This does not alter the authors’ adherence to all the PLOS ONE policies on sharing data and materials. There are no patents, products in development or marketed products to declare."

Please confirm that this does not alter your adherence to all PLOS ONE policies on sharing data and materials, by including the following statement: "This does not alter our adherence to PLOS ONE policies on sharing data and materials.” (as detailed online in our guide for authors http://journals.plos.org/plosone/s/competing-interests). If there are restrictions on sharing of data and/or materials, please state these. Please note that we cannot proceed with consideration of your article until this information has been declared.

Please include your updated Competing Interests statement in your cover letter; we will change the online submission form on your behalf.

**Author response**: We confirm and update our competing interest statement in our cover letter to include the statement exactly as stated above.

4. PLOS requires an ORCID iD for the corresponding author in Editorial Manager on papers submitted after December 6th, 2016. Please ensure that you have an ORCID iD and that it is validated in Editorial Manager. To do this, go to ‘Update my Information’ (in the upper left-hand corner of the main menu), and click on the Fetch/Validate link next to the ORCID field. This will take you to the ORCID site and allow you to create a new iD or authenticate a pre-existing iD in Editorial Manager. Please see the following video for instructions on linking an ORCID iD to your Editorial Manager account: <https://www.youtube.com/watch?v=_xcclfuvtxQ>

**Author response**: We have ensured that the corresponding author has an ORCID iD, and that it is validated in the Editorial Manager

5. In your Data Availability statement, you have not specified where the minimal data set underlying the results described in your manuscript can be found. PLOS defines a study's minimal data set as the underlying data used to reach the conclusions drawn in the manuscript and any additional data required to replicate the reported study findings in their entirety. All PLOS journals require that the minimal data set be made fully available. For more information about our data policy, please see http://journals.plos.org/plosone/s/data-availability.

Upon re-submitting your revised manuscript, please upload your study’s minimal underlying data set as either Supporting Information files or to a stable, public repository and include the relevant URLs, DOIs, or accession numbers within your revised cover letter. For a list of acceptable repositories, please see http://journals.plos.org/plosone/s/data-availability#loc-recommended-repositories. Any potentially identifying patient information must be fully anonymized.

Important: If there are ethical or legal restrictions to sharing your data publicly, please explain these restrictions in detail. Please see our guidelines for more information on what we consider unacceptable restrictions to publicly sharing data: http://journals.plos.org/plosone/s/data-availability#loc-unacceptable-data-access-restrictions. Note that it is not acceptable for the authors to be the sole named individuals responsible for ensuring data access.

We will update your Data Availability statement to reflect the information you provide in your cover letter.

**Author response**: We have included our study’s minimal underlying data set as a supporting information file (File: S1_Dataset).

6. Please upload a new copy of all Figures as the detail is not clear. Please follow the link for more information: https://blogs.plos.org/plos/2019/06/looking-good-tips-for-creating-your-plos-figures-graphics/" <https://blogs.plos.org/plos/2019/06/looking-good-tips-for-creating-your-plos-figures-graphics/>

**Author response**: Thank you for this reference. We found the PACE tool incredibly useful for evaluating figures against your platform requirements. All new figures are flattened with 300-600 DPI resolution and LZW compression.

7. Please review your reference list to ensure that it is complete and correct. If you have cited papers that have been retracted, please include the rationale for doing so in the manuscript text, or remove these references and replace them with relevant current references. Any changes to the reference list should be mentioned in the rebuttal letter that accompanies your revised manuscript. If you need to cite a retracted article, indicate the article’s retracted status in the References list and also include a citation and full reference for the retraction notice.

**Author response**: We provided an additional reference to the SSQ questionnaire and two additional references to R packages that were used as part of suggested analyses by a reviewer. We also updated a broken link in reference #27. All other references are complete and correct. All changes are mentioned in our rebuttal letter above.

**Additional Editor Comments:**

Add colors to figures 1 and 2

**Author response**: Figures 1 and 2 have been revised to include color.

**Review Comments to the Author**

**Reviewer #1**:

**Comment 1**: Excellent article regarding the need to evaluate whether CROS or hearing aids are best for patients with unilateral CI and hearing loss in the contralateral ear. It is a debatable question as it depends on the patients perception but the authors aimed to assess various aspects including a questionnaire, and the inclusion criteria were adequate.

**Author response**: We appreciate your thoughtful review and comment. The debatable nature of the CROS or HA argument informed our intention to comprehensively address these various aspects, in addition to speech recognition outcomes.

**Reviewer #2**:

Comment 1: The manuscript by Lively et al. is a well written report about the benefits of bimodal listening, aiding the contralateral ear in unilateral CI patients. The authors show significant improvement in speech in noise and lateralization in most patients when a haring aid or CROS device were implemented in the non-implanted ear. They also discussed the limitation of the study regarding small sample size and previous use of hearing aid devices.

Overall, methodology is clear and could be replicated, the data and figures are well laid-out but could benefit from showing plots including self-reported information. Statistics are well reported in the manuscript, but the analysis of the slopes is not appropriate to the data considering the small sample size.

**Author response**: Thanks for the suggestion! We have generated Fig 3, which includes a summary of individual data from the subjective assessments, and added the following figure caption:

*“Each boxplot represents the distribution of the average response from each of the 18 participants in the communication needs domain (panel A) and the spatial awareness needs domain (panel B) at baseline and after chronic CROS and HA use. The horizontal line within each boxplot represents the median, while the upper and lower boundaries of the boxplot indicate the interquartile range (IQR). The whiskers extend to the highest and lowest average within 1.5 times the IQR. Data points beyond this range are considered outliers. The "x" shape represents the average response collapsed across all participants for a particular domain. The colored symbols represent the responses from participant 2 (red square), participant 3 (orange triangle), and participant 20 (yellow circle) who opted to keep the CROS device at the end of the study, while the gray filled circles represent the individuals who chose to keep the HA at the end of the study.”*

Additional commentary on edits regarding the slope analyses are addressed under comment 4.

**Comment 2:** Participants were randomized to either HA or CROS group for phase 1 and then fitted with the other device on phase 2. Was there any effect of the starting group on speech/localization or self-reported measures?

**Author response**: Linear mixed-effects models were used to analyze the effect of the starting group (CROS or HA) on the results from the questionnaires, lateralization, and speech recognition tasks. The analysis revealed no statistically significant effect of the starting device group on any outcome measure. We have added these findings to the results section.

**Comment 3:** Although questionnaires were developed with the assistance of experts, could you have used other questionnaires used by other research/clinical groups? This helps when comparing the outcomes of studies like this one, improving the impact of the evidence.

**Author response**: While developing our questionnaire, we did consider the option of utilizing established instruments such as the SSQ. However, recognized nuances intrinsic to the work at hand presented unique challenges that existing validated questionnaires couldn't fully address. One of the central aspects of our study was the communication needs assessment. Our questionnaires were designed to explore how each communication device met (or did not) those unique needs and added a dimension that we are unaware any currently established questionnaires capture comprehensively.

We want to acknowledge the inherent limitation of not employing a validated questionnaire. In our revised manuscript, we have added the following to the limitations section:

*“An important consideration in our study was the selection of a suitable questionnaire to assess the communication needs and the extent to which the evaluated hearing devices met those needs. While established and validated questionnaires, such as the SSQ [42], are commonly employed in research endeavors of this nature, we chose to develop a novel questionnaire tailored to the specifics of our research objectives.*

*The decision not to adopt an existing validated questionnaire is acknowledged as a limitation of our study, as validated questionnaires provide a standardized framework for data collection and facilitate inter-study comparisons. We recognize that future studies in this domain may benefit from considering a dual-method approach, incorporating both established validated instruments and context-specific questionnaires to strike a balance between standardization and the uniqueness of research objectives.”*

**Comment 4:** According with other studies and your results, investigating the slope of the hearing loss could give more information to help the decision of which device to select in order to provide more benefit to the patient. However, the statement of grater benefit = shallower slopes in all groups is not clear as showed in Figure 4, whit only 4 subjects in the CB group and high dispersion. The statement is clearer in the HA group. More important could be reporting the slope (using correlation test) of the fitted line in each condition than using ANOVA for comparing the group, which could be useful with a bigger sample size.

**Author response**: We agree that sample size is indeed a limitation of this study. Because of this we have omitted the exploratory analyses on the slopes. We also acknowledge the ambiguity in our statement regarding the relationship between slope and benefit due to the small sample size in the CB group. To clarify this point, we have toned down the language and provided additional context in the manuscript that includes commentary on the dispersion observed in Figure 4 for the CB group and small n in this group that limits our conclusions without more data.

**Comment 5:** Please report the final device selection in the results section.

**Author response**: We have added an “End of study device preference” section to the results and included the following statement:

*“A total of 18 participants were involved in the research investigation. After the study, 15 participants decided to retain the Hearing Aid (HA) they used throughout the study. Of the three individuals who opted to keep the CROS device, two were from the CB group, and one was from the EB group.”*

**Comment 6:** I do agree with the authors and evidence that the self-reported measures are important when selecting an intervention like the ones reported here. I suggest showing the association of data from questionnaires, behavioral measures, and final device selection. This could be helpful to understand the complexity of the decision-making process, specially because most of the participants of this study chose HA over CROS device for the un-implanted ear even if the later one showed to be more beneficial for some of them in speech in noise and lateralization of sounds.

**Author response**: In our study, only a limited number of participants opted for the CROS device at the end of the study, which restricts our ability to analyze these relationships and reveal any trends or patterns. However, we agree that such an analysis would provide valuable insights into the decision-making process of participants regarding device selection. Recognizing the importance of these relationships, the previous version of this manuscript discussed the results from the questionnaires in the context of final device preference. Additionally, in this revised version, all figure captions highlight individuals who kept the CROS to contrast them from the participants who kept the HA for greater transparency.

**Comment 7:** The results of this work also show patients with no benefit (ie. better performance with CI-only) in comparison with HA and/or CROS devices. This issue should also be discussed in here (Ln 330-331). Only reporting the benefit with HA or CROS or equivalent benefit is bias.

**Author response**: This is an excellent point. We have added to the discussion some commentary on individuals who experienced declines in speech and lateralization task performance with the addition of a contralateral device.

**Minor comment 1:** ID of participants on table 1 must match the ID number used in the plots. For example, in Fig 1 and 2 the authors used subject ID’s from 1 to 18, but in Table 1 and Fig 3, they used S1 to S21.

**Author response**: This has been addressed. The subject IDs in Figures 1, 2, and 4 now match with the subject IDs presented in Table 1.

**Minor comment 2:** Figures and statistics: it is necessary to include statistically significant and non-significant differences in the figures (e.g., Fig 1 and 2).

**Author response**: We have added statistically significant and non-significant differences to figures 1 and 2.

**Minor comment 3**: Figure 2: “*” representing median data for CI+CROS condition is missed. Please check and include the symbol.

**Author response**: Thank you for noticing this. We added this symbol because the median fell exactly on the y-grid line, and the median could not be seen in the B&W version. Since updating the figures to color, we have removed the “*” as the median is now visible.

**Minor comment 4:** Figure 3: Please show (eg. Shaded area) or describe the fitting range for the definition of “aidable threshold”. Also, we suggest including this information in the text, line 262-263.

**Author response**: Note: This is now Figure 4. The initial version of the manuscript describes an “aidable threshold” as one that falls within the fitting range of the Phonak Link M hearing aid. Per the reviewer's suggestion, we have added to the text to clarify the fitting range of the Link M in this section that now states, “A threshold was defined as aidable if it fell within the fitting range of Phonak’s Naída Link M BTE, which is capable of fitting moderate to profound hearing loss of all audiometric configurations.” In addition, we have overlaid the fitting range of the hearing aid onto Fig 4 and updated the figure caption to include the text….” The shaded region indicates the fitting range of the Phonak Link M hearing aid.”

Minor comment 5: Line 350: replace , with .

**Author response**: The text now reads, “Therefore, the current study included baseline and chronic use subjective questionnaires to assist with evidence-based, clinic-based outcome measures to help guide decisions in clinical practice. This information was particularly helpful for this subset population of unilateral CI users where the difference in speech recognition performance in noise was not statistically different between listening conditions, yet there was a clear device preference.”
